# Clinicopathological characteristics and treatment outcomes of biliary neuroendocrine carcinoma: a single-center case series and literature review

**DOI:** 10.3389/fonc.2026.1745019

**Published:** 2026-02-11

**Authors:** Zhipeng Liu, Faji Yang, Yijie Hao, Yuan Zhao, Shizhe Zhang, Zheyu Niu, Xin Wang, Liyang Sun, Huaqiang Zhu, Fengyun Cui, Jun Lu, Hengjun Gao, Xu Zhou

**Affiliations:** 1 Department of Pancreatobiliary Surgery, Shandong Provincial Hospital Affiliated to Shandong First Medical University, Jinan, China; 2 Department of Pathology, Shandong Provincial Hospital Affiliated to Shandong First Medical University, Jinan, China

**Keywords:** biliary neuroendocrine carcinoma, clinicopathological features, immunohistochemistry, prognosis, surgical resection

## Abstract

**Objective:**

To investigate the clinicopathological characteristics, treatment strategies, and prognostic outcomes of biliary neuroendocrine carcinoma (Biliary NEC), and to review the relevant literature to provide further evidence for the clinical management of this rare malignancy.

**Methods:**

We retrospectively analyzed the clinical data of nine patients who underwent surgical resection and were pathologically diagnosed with biliary NEC at Shandong Provincial Hospital between May 2012 and October 2025. Clinical manifestations, imaging findings, surgical procedures, histopathological and immunohistochemical results (Syn, CgA, CD56, Ki-67), and follow-up outcomes were collected. Overall survival (OS) was estimated using the Kaplan–Meier method. Additionally, published case reports and case series from 2020 onward were reviewed for comparison.

**Results:**

Among the nine patients (3 males and 6 females; median age, 65 years; range, 57–77 years), the primary tumor sites included the gallbladder (n = 4), hilar bile duct (n = 3), and distal bile duct (n = 2). The main presenting symptoms were abdominal discomfort (n = 6) and jaundice (n = 3). All tumors were poorly differentiated NECs, comprising six small-cell and two large-cell types, with Ki-67 indices ranging from 30% to 80% (median, 70%). Immunohistochemistry showed Syn (+), CgA (±), and CD56 (±), with partial expression of SSTR2. All patients underwent curative-intent resection (R0 in 8 and R1 in 1), and four received systemic chemotherapy initiated after documented tumor recurrence. The median follow-up duration was 649 days (range, 67–953 days), and the median OS was 649 days (95% CI: 85–1213 days). A review of the literature revealed that systemic chemotherapy, particularly platinum-based regimens, was associated with longer median survival (18 vs. 9 months).

**Conclusions:**

Biliary NEC is a rare and highly aggressive malignancy with nonspecific clinical manifestations, and its diagnosis relies primarily on histopathological and immunohistochemical evaluation. Surgical resection remains the cornerstone of treatment, while systemic chemotherapy may provide additional survival benefit in selected patients. A high Ki-67 index and lymph node metastasis have been consistently reported as adverse prognostic indicators in biliary neuroendocrine carcinoma, primarily based on evidence from previously published studies. Given the limited sample size of the present case series, our observations should be interpreted as descriptive rather than statistically conclusive. Future multicenter studies incorporating molecular and immunologic profiling are warranted to clarify the biological behavior of biliary NEC and to optimize individualized therapeutic strategies.

## 1 Introduction

Biliary neuroendocrine carcinoma (Biliary NEC) is a rare and highly aggressive malignant tumor originating from neuroendocrine cells within the biliary system. Its incidence is extremely low, accounting for less than 1% of all gastroenteropancreatic neuroendocrine neoplasms (GEP-NENs), and it most commonly arises in the gallbladder or the common bile duct (1). Because neuroendocrine cells are sparsely distributed in the biliary tract (2), the pathogenesis of Biliary NEC remains poorly understood. Several hypotheses have been proposed, suggesting that it may originate from neuroendocrine metaplasia of the biliary epithelium, clonal evolution of adenocarcinoma, or aberrant differentiation of pluripotent stem cells (3, 4).

Clinically, Biliary NEC lacks specific manifestations. The most common presenting symptoms include upper abdominal pain, obstructive jaundice, and unexplained weight loss. Its imaging features often mimic those of cholangiocarcinoma or gallbladder adenocarcinoma, leading to a high rate of preoperative misdiagnosis (5, 6). Common tumor markers such as CA19-9, CEA, and CA125 are frequently normal or only mildly elevated (7). Therefore, definitive diagnosis typically depends on postoperative histopathological and immunohistochemical findings, including positive staining for markers such as synaptophysin (Syn), chromogranin A (CgA), and CD56 (5, 8).

Histologically, Biliary NEC represents a poorly differentiated and high-grade neuroendocrine neoplasm, which can be classified into small-cell (SCNEC) and large-cell (LCNEC) types. It differs fundamentally from well-differentiated NET G3 in both morphology and biological behavior (4, 9). The characteristic pathological features include marked nuclear atypia, extensive necrosis, frequent mitoses, and a high Ki-67 proliferation index (typically >50%), indicating a highly aggressive biological phenotype (10).

Currently, there is no standardized treatment guideline for Biliary NEC. For patients with resectable disease, radical surgery remains the mainstay of therapy and offers the best chance for long-term survival. Systemic treatment strategies are generally extrapolated from those used for small-cell lung cancer and extrapulmonary NECs, with platinum-based doublet regimens—such as etoposide plus cisplatin (EP) or irinotecan plus cisplatin—being commonly adopted (11, 12). However, most available studies consist of isolated case reports or small case series, and robust evidence regarding surgical indications, systemic chemotherapy strategies, and long-term prognosis remains scarce.

Given its rarity, diagnostic challenges, and therapeutic heterogeneity, Biliary NEC continues to pose a significant clinical challenge. This study retrospectively analyzed patients with pathologically confirmed Biliary NEC who underwent surgical resection at our institution between 2012 and 2025. We aimed to systematically summarize their clinicopathological characteristics, treatment patterns, and survival outcomes, and to compare our findings with those reported in the literature, thereby providing additional evidence for clinical decision-making in this uncommon malignancy.

## 2 Materials and methods

### 2.1 Patients and data collection

This was a single-center, retrospective case series. Patients who underwent surgical resection and were pathologically diagnosed with biliary neuroendocrine carcinoma (Biliary NEC) at Shandong Provincial Hospital between May 2012 and October 2025 were included. Clinical data were obtained from the hospital’s electronic medical record and pathology databases. Two independent investigators reviewed all clinical information to ensure completeness and accuracy.

This study was conducted as a retrospective analysis using anonymized patient data and did not involve any direct interventions. According to the regulations of the Ethics Committee of Shandong Provincial Hospital, separate ethical approval was waived for this type of analysis.

### 2.2 Preoperative assessment and treatment planning

All patients underwent comprehensive preoperative evaluation, including complete blood count, liver and renal function tests, serum tumor markers, chest CT, contrast-enhanced upper abdominal CT, and magnetic resonance cholangiopancreatography (MRCP). In selected cases, ^18F-fluorodeoxyglucose positron emission tomography/computed tomography (^18F-FDG PET/CT) was performed to assess metabolic activity and detect possible metastases.

Treatment strategies were determined through multidisciplinary team (MDT) discussions. Imaging examinations were used to evaluate tumor location, size, relationship with adjacent vasculature and biliary structures, and the presence of distant metastases. Patients without evidence of distant spread and with resectable disease were considered candidates for curative-intent resection. The extent of surgery was determined by tumor site and invasion range, with hepatic or pancreatic resection performed when necessary. For patients presenting with obstructive jaundice, percutaneous or endoscopic biliary drainage was conducted preoperatively to improve hepatic function.

### 2.3 Follow-up

All patients were followed up systematically through outpatient visits and telephone contact, with the last follow-up performed in October 2025. Follow-up evaluations included imaging and laboratory examinations. Tumor recurrence was confirmed by radiologic or histopathologic evidence. Overall survival (OS) was defined as the interval between the date of surgery and death from any cause or the last follow-up.

### 2.4 Statistical analysis

All statistical analyses were performed using R software (version 4.2.1). Continuous variables were expressed as median (range) or mean ± standard deviation (SD), while categorical variables were summarized as counts and percentages. Survival analysis was conducted using the Kaplan–Meier method to estimate median OS and 95% confidence intervals (CIs). Due to the limited sample size, multivariate Cox regression analysis was not performed. A two-sided *P* value < 0.05 was considered statistically significant.

### 2.5 Literature review methodology

A focused literature review was conducted using PubMed and Web of Science databases. The search was limited to articles published between January 2020 and October 2025. Search terms included “biliary neuroendocrine carcinoma”, “gallbladder neuroendocrine carcinoma”, “extrahepatic bile duct neuroendocrine carcinoma”, and related combinations.

Only English-language case reports and case series with histopathologically confirmed biliary neuroendocrine carcinoma were included. Reviews without individual patient data, conference abstracts, and studies lacking essential clinicopathological or survival information were excluded. Two authors independently screened titles, abstracts, and full texts, and discrepancies were resolved through discussion and consensus.

## 3 Results

### 3.1 Patient selection and overall characteristics

**Figure 1:**
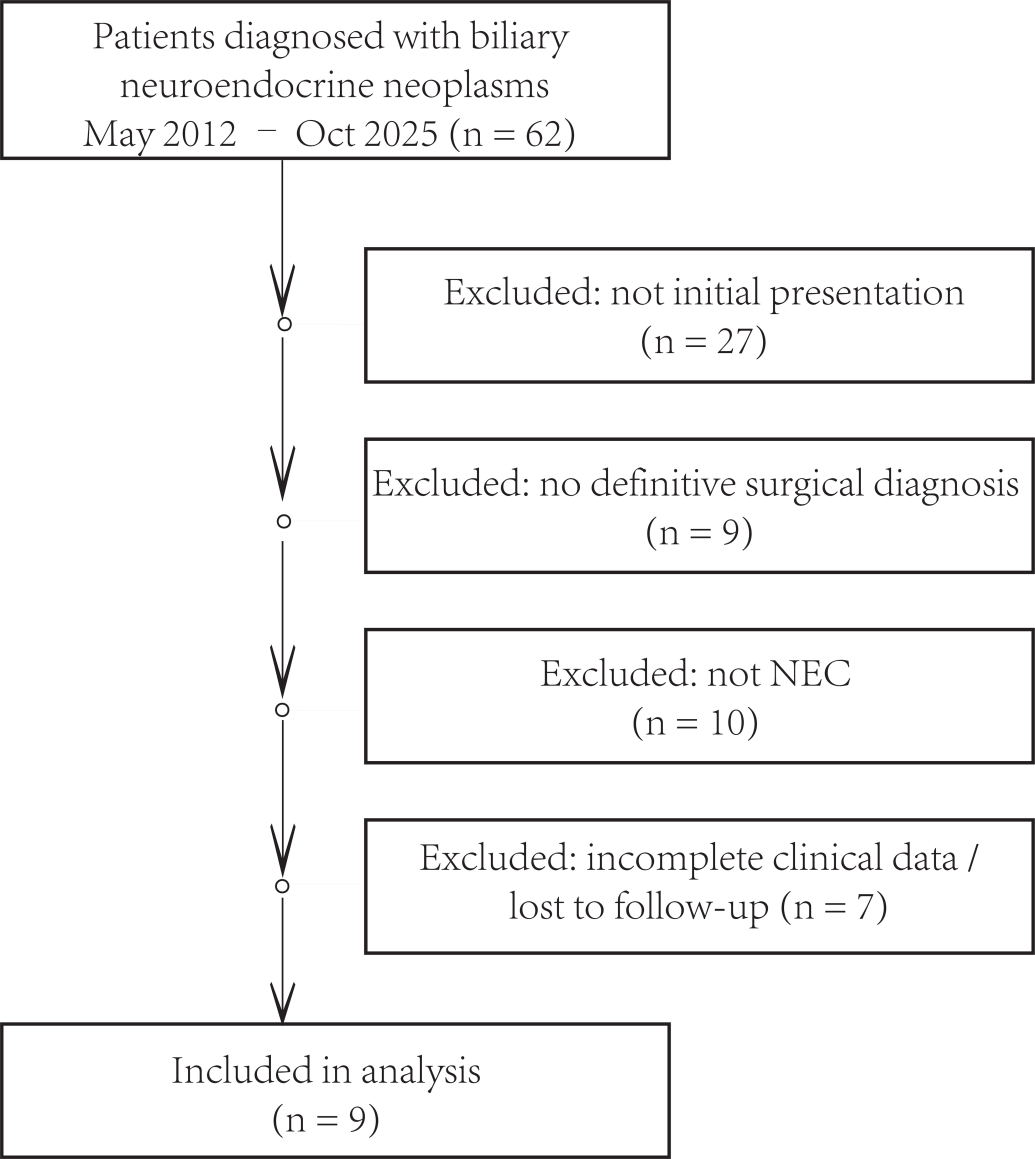
Flow diagram of patient selection.

Between May 2012 and October 2025, a total of 62 patients with suspected or confirmed biliary neuroendocrine neoplasms were identified at our institution. After excluding patients who were not first-time visitors (n = 27), those without surgical pathological confirmation (n = 9), cases with non-neuroendocrine carcinoma histology (n = 10), and those with incomplete clinical data or loss to follow-up (n = 7), nine patients with pathologically confirmed biliary neuroendocrine carcinoma (Biliary NEC) were finally included in this study (**Figure 1**). All patients underwent surgical resection and were confirmed by histopathological and immunohistochemical analyses.

### 3.2 Clinical and pathological characteristics

The clinical and baseline characteristics of the nine patients are summarized in **Table 1**. There were three men and six women, with a median age of 65 years (range, 57–77 years). The primary tumor sites included the gallbladder (n = 4), hilar bile duct (n = 3), and distal bile duct (n = 2). The main presenting symptoms were abdominal discomfort (n = 6) and jaundice (n = 3), while one lesion was detected incidentally during routine physical examination.

**Table 1:** Baseline clinical characteristics of nine patients with biliary neuroendocrine carcinoma.

Case no.	Age	Sex	Primary site	Presenting symptoms	CEA (U/ml)	CA199 (U/ml)	CA125 (U/ml)
1	63	Male	Distal bile duct	Abdominal discomfort+Jaundice	N	N	48.26
2	77	Male	Distal bile duct	Abdominal discomfort	N	N	821
3	61	Male	Gallbladder	Physical examination	N	N	N
4	57	Female	Gallbladder	Abdominal discomfort	N	N	N
5	72	Female	Gallbladder	Abdominal discomfort	N	N	N
6	74	Male	Gallbladder	Abdominal discomfort+Jaundice	N	N	176.5
7	65	Female	Hilar bile duct	Jaundice	N	N	40.3
8	67	Female	Hilar bile duct	Jaundice	N	33.5	112
9	57	Female	Hilar bile duct	Abdominal discomfort	N	N	N

Preoperative serum tumor markers revealed that only one patient had a mildly elevated CA19–9 level, whereas CEA and CA19–9 levels were within normal ranges in the remaining cases. Four patients presented with elevated CA125 (48.26–821 U/mL).

**Figure 2:**
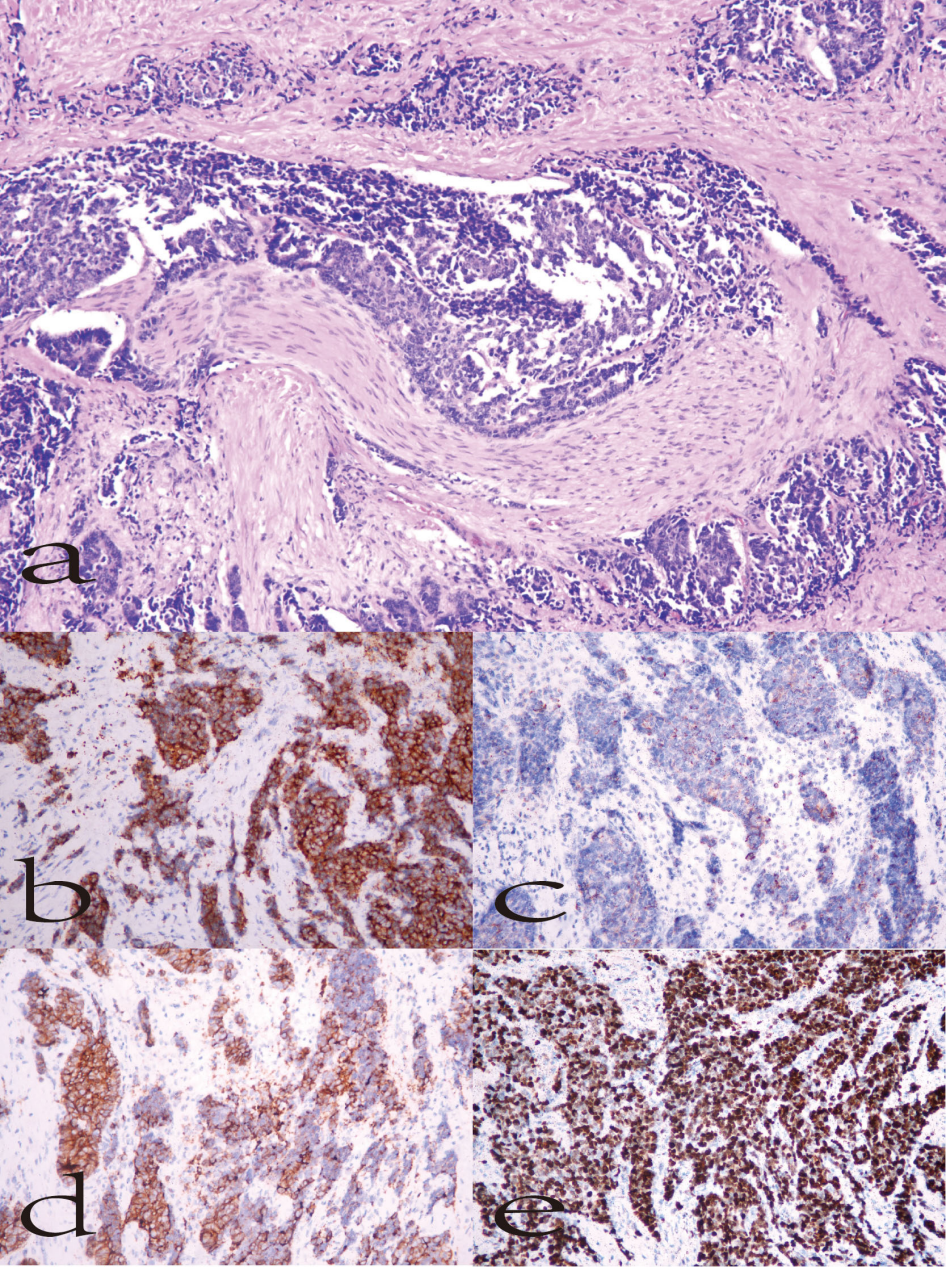
Representative histopathological and immunohistochemical findings of biliary neuroendocrine carcinoma. **(a)** HE staining (200×), showing diffuse tumor cell arrangement with nuclear atypia and necrosis. **(b)** Syn positivity; **(c)** CgA positivity; **(d)** CD56 positivity; **(e)** Ki-67 positivity (~70%).

Representative histological and immunohistochemical findings are shown in **Figure 2**. Hematoxylin–eosin (HE) staining demonstrated diffusely arranged tumor cells with marked nuclear atypia and necrosis. Immunohistochemistry revealed positive staining for Syn, CgA, and CD56, and a Ki-67 proliferation index of approximately 70%, consistent with a poorly differentiated, highly proliferative neuroendocrine phenotype.

### 3.3 Treatment characteristics

All nine patients underwent surgical resection, including eight R0 and one R1 resection. Surgical procedures included pancreaticoduodenectomy with lymph node dissection (PD + LND) in two cases, cholecystectomy with hepatic segment IVb/V resection and lymphadenectomy (Cho + LND + IVb/V) in three cases, and common bile duct resection with biliary reconstruction (Cho + LND + BDR) in four cases. No distant metastases were detected intraoperatively.

Postoperative histopathology confirmed all tumors as poorly differentiated NECs, consisting of six small-cell (SCNEC) and two large-cell (LCNEC) types; one case was not subclassified. Ki-67 indices ranged from 30% to 80% (median, 70%). Immunohistochemistry revealed Syn (+), CgA (±), and CD56 (±) expression.

**Figure 3:**
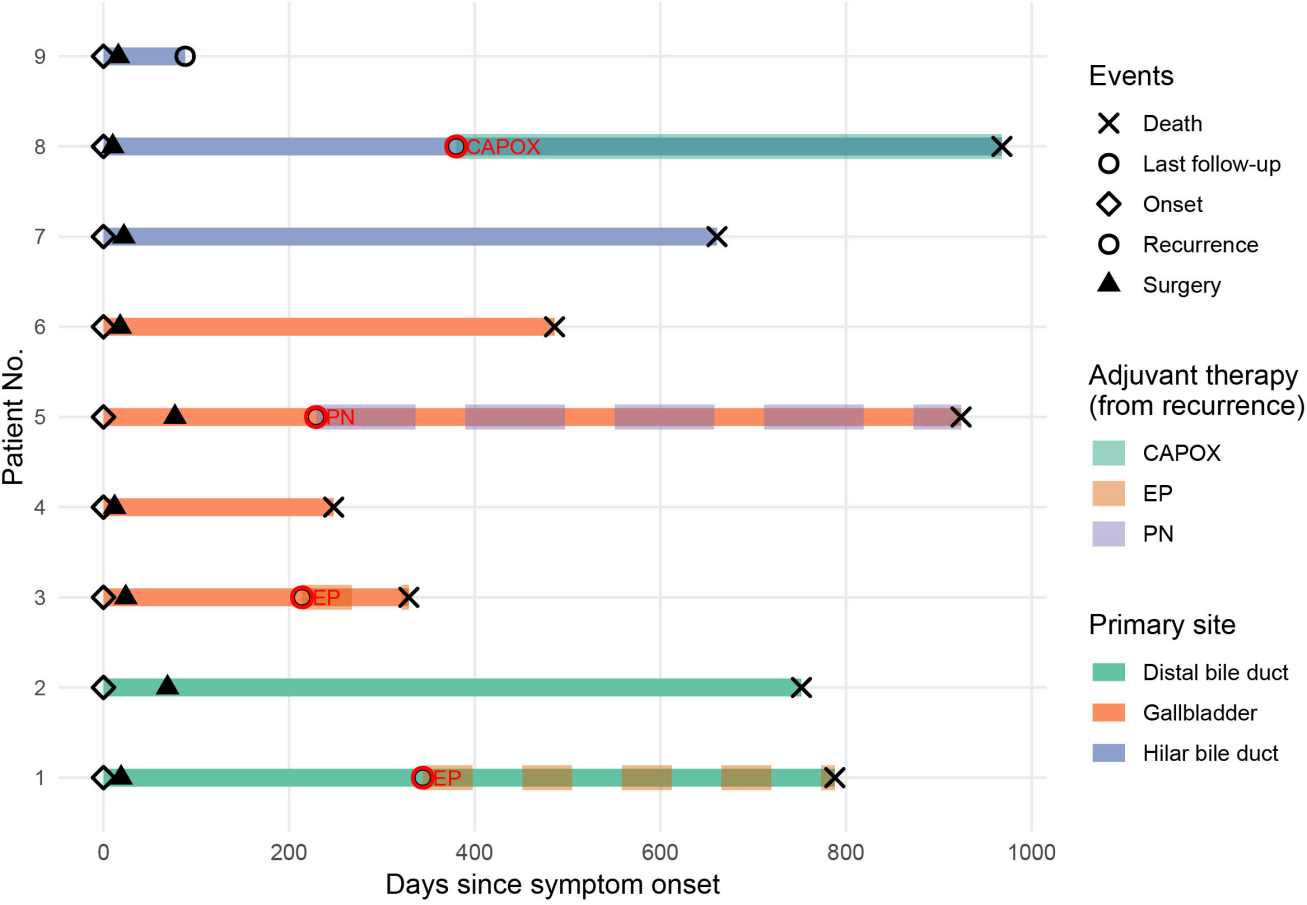
Swimmer plot illustrating the clinical course and treatment timeline of nine patients with biliary NEC.

According to the 8th edition of the AJCC staging system, two patients were classified as stage I, three as stage IIA, three as stage IIB, and one as stage IIIB. Lymph node metastasis was identified in four patients (44.4%). Despite a high R0 resection rate (88.9%), most patients presented with advanced T stage and/or nodal involvement, indicating a substantial tumor burden at diagnosis. Detailed surgical and pathological findings are presented in **Table 2**. Postoperative management was determined based on pathological stage, Ki-67 index, and patient performance status. Four patients received systemic chemotherapy after documented tumor recurrence, mainly consisting of platinum-based regimens. **Figure 3** illustrates the clinical course and treatment timeline of the nine patients.

**Table 2:** Surgical procedures, TNM staging, and pathological characteristics of nine patients with biliary neuroendocrine carcinoma.

Surgical and prognostic								
Case no.	Surgical procedure	Margin	TNM stage	AJCC stage	Post-recurrence systemic chemotherapy	Status		Survival (days)
1	PD+LND	R0	T2aN0M0	IIA	EP	Dead		759
2	PD+LND	R0	T1N1M0	IIB	None	Dead		674
3	Cho + LND + IVb, V	R0	T3N1M0	IIIB	EP	Dead		302
4	Cho + LND + IVb, V	R1	T3N1M0	IIIB	None	Dead		230
5	Cho + LND + IVb, V	R0	T2aN1M0	IIIB	PN	Dead		781
6	Cho + LND + IVb, V	R0	T2aN0M0	IIA	None	Dead		465
7	Cho + LND + BDR	R0	T2aN0M0	IIA	None	Dead		624
8	Cho + LND + BDR	R0	T1N0M0	I	CAPOX	Dead		953
9	Cho + LND + BDR	R0	T1N0M0	I	None	Alive		67

Clinicopathological characteristics								
Case no.	Tumor Location	Ki-67(%)	Histology	Syn		CgA	CD56	
1	Distal bile duct	70	SCNEC	+		–	+	
2	Distal bile duct	80	SCNEC	+		+	+	
3	Gallbladder	80	LCNEC	+		+	+	
4	Gallbladder	30	SCNEC	+		+	+	
5	Gallbladder	70	SCNEC	+		+	+	
6	Gallbladder	80	/	+		+	+	
7	Hilar bile duct	70	LCNEC	+		+	+	
8	Hilar bile duct	60	SCNEC	+		–	/	
9	Hilar bile duct	80	/	+		+	+	

**Figure 4:**
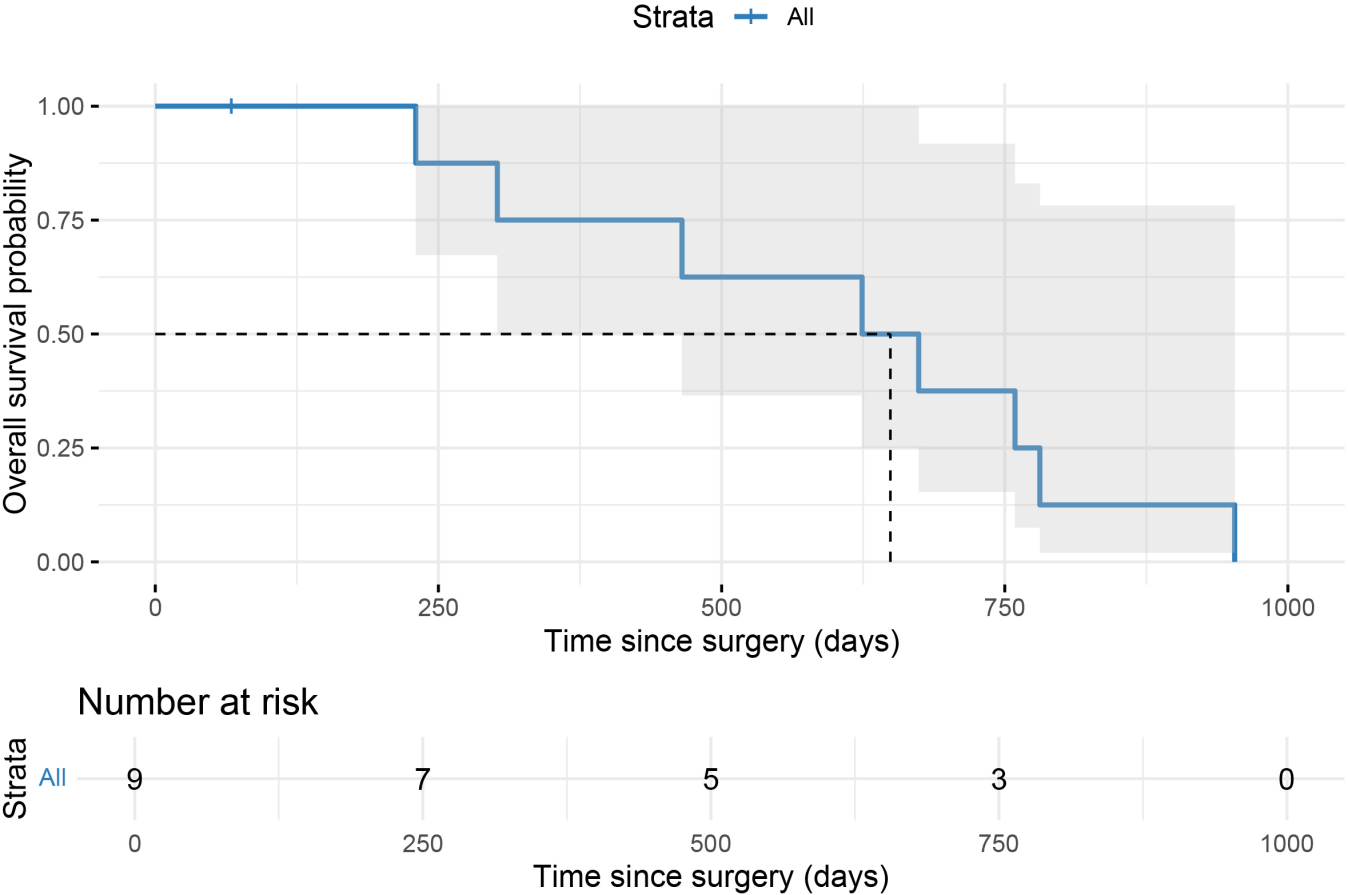
Kaplan–Meier overall survival curve for patients after surgery.

By October 2025, all nine patients had completed follow-up. The median follow-up duration was 649 days (range, 67–953 days). Eight patients died during follow-up, and one patient (Case 9) remained alive. Kaplan–Meier analysis showed a median overall survival (OS) of 649 days (95% confidence interval, 85–1213 days) (**Figure 4**).

### 3.4 Literature review and summary

To further characterize the clinical features of biliary neuroendocrine carcinoma (BNEC), 15 published case reports and case series since 2020 were reviewed, encompassing 17 patients (**Table 3**). Reported cases involved both sexes equally, with ages ranging from 29 to 83 years (median, 65 years). The most frequent primary sites were the gallbladder, distal bile duct, and hilar bile duct. The predominant symptoms were abdominal pain and jaundice, whereas a few patients presented with cachexia or were incidentally detected.

**Table 3:** Summary of previously reported cases of biliary neuroendocrine carcinoma.

Author	Age	Sex	Presenting symptoms	Primary site	Surgical procedure	Margin	Histology	Ki-67(%)	Syn	CgA	CD56	Post-recurrence systemic chemotherapy	OS(months)	Status
Rennie (13)	83	F	Abdominal discomfort	Gallbladder	Cho + LND + IVb, V	R0	SCNEC	70	+	+	+	EC	/	/
Ren Xu (14)	70	F	Abdominal discomfort	Gallbladder	Cho	R0	LCNEC	80	+	+	/	/	30	Dead
	64	F	Abdominal discomfort	Gallbladder	Cho + LND	R0	LCNEC	80	+	+	/	/	/	/
Sah (15)	65	F	Abdominal discomfort	Gallbladder	Cho + LND + IVb, V	R0	LCNEC	/	/	/	/	/	/	/
Liao Yong (16)	65	M	Physical examination	Gallbladder	Cho + LND + RHP	R0	/	50	+	+	+	EP	12	Alive
	66	M	Abdominal discomfort	Gallbladder	Cho + LND + IVb, V	R0	LCNEC	80	/	+	+	EP	14	Alive
Sugita H (17)	62	M	Jaundice	Distal bile duct	SSPPD	R0	SCNEC	80	+	+	/	/	4	Alive
Yao (18)	52	M	Abdominal discomfort	Gallbladder	Cho + LND	R0	/	70	+	+	+	EP	16	Alive
Altiti (19)	36	F	Jaundice	Hilar bile duct	BDR + Cho + RYHJ	R0	/	/	+	+	/	/	12	Alive
Tamaki (20)	69	F	Abdominal discomfort	Distal bile duct	SSPPD	R0	LCNEC	30	+	+	+	EP	64	Alive
	79	M	Marasmus	Distal bile duct	SSPPD	R0	MINEN	30	+	+	+	/	/	Alive
Jevdokimov (21)	42	F	Jaundice	Hilar bile duct	BDR + Cho + RYHJ	R0	/	80	+	+	–	FOLFOX6	/	Alive
Liu Sulai (22)	65	F	Jaundice	Hilar bile duct	BDR + Cho + RYHJ	R0	MINEN	70	+	+	/	/	34	Alive
Nakamura (23)	74	M	Marasmus	Distal bile duct	PPPD	R0	SCNEC	95	/	+	+	EC	67	Alive
Shim (24)	64	M	Jaundice	Distal bile duct	PPPD	R0	SCNEC	60	+	+	+	EP	36	Alive
Kiya (25)	29	F	Abdominal discomfort	Distal bile duct	PPPD	R0	LCNEC	90	+	+	+	PI	16	Alive
Chen Fei (26)	62	F	Abdominal discomfort	Distal bile duct	PD	R0	LCNEC	50	+	+	/	/	24	Dead

Most tumors were poorly differentiated, including large-cell and small-cell subtypes, with Ki-67 indices of 30–95% and frequent positivity for Syn, CgA, and CD56. Approximately half of the patients received platinum-based systemic chemotherapy, and their median overall survival was longer than that of patients without chemotherapy (approximately 20 months vs. 10 months).

## 4 Discussion

Biliary neuroendocrine carcinoma (Biliary NEC) is an extremely rare and highly aggressive malignancy, accounting for approximately 0.5% of all neuroendocrine neoplasms (NENs) (27). Due to its low incidence, nonspecific clinical manifestations, and rapid progression, most patients are diagnosed at an advanced stage and consequently miss the optimal window for curative treatment (28–30). Therefore, the overall prognosis of Biliary NEC remains dismal. Although the number of reported cases has gradually increased in recent years owing to advances in immunohistochemistry and imaging technology, large-scale studies are still lacking, and the understanding of this disease remains limited. In this study, we retrospectively analyzed nine cases of pathologically confirmed Biliary NEC treated at our institution over the past decade and compared our findings with previous reports to summarize the clinicopathological characteristics, therapeutic strategies, and prognostic outcomes, thereby enriching current evidence on this rare malignancy.

Consistent with previous studies, patients with Biliary NEC in our cohort had a median age of approximately 60 years and a slight female predominance. Histologically, most tumors were poorly differentiated, either small-cell neuroendocrine carcinoma (SCNEC) or large-cell neuroendocrine carcinoma (LCNEC), with Ki-67 indices commonly exceeding 50%. The clinicopathological and immunophenotypic features in our study—positive expression of Syn, CgA, and CD56—were largely in line with previous observations. The median overall survival (OS) in our series was 649 days (approximately 21 months), comparable to the 25 months reported by Ayabe et al. (31) and 23 months by Zhou et al. (30). However, those studies included some well-differentiated NENs, which may account for their relatively better outcomes (32, 33). In contrast, Karim et al. (34), analyzing 278 cases of gallbladder NEC from the SEER database, reported a median OS of only 9.8 months—significantly shorter than in our study. This discrepancy may be attributed to the fact that all patients in our cohort underwent curative-intent resection, and several received systemic chemotherapy.

Accumulating evidence suggests that systemic chemotherapy, particularly platinum-based regimens, may improve survival in patients with biliary neuroendocrine carcinoma (35–38). Chu et al. (27) reported that postoperative systemic chemotherapy prolonged overall survival by approximately 6.7 months compared with surgery alone, while Lee et al. (39) identified adjuvant chemotherapy as an independent prognostic factor (P = 0.007). Similarly, in our study, patients who received systemic chemotherapy showed a trend toward longer survival compared with those who did not; however, this observation should be interpreted cautiously given the limited sample size and descriptive nature of the analysis.

Although R0 resection is traditionally regarded as a prerequisite for long-term survival in biliary malignancies, accumulating evidence indicates that, in biliary neuroendocrine neoplasms (NENs), margin-negative surgery alone is often insufficient to overcome the intrinsically aggressive tumor biology. Recent population-based and multi-institutional studies have shown that patients with biliary NENs are frequently diagnosed at advanced stages and exhibit high proliferative indices, nodal involvement, or distant metastases, ultimately resulting in poor median overall survival despite relatively high rates of R0 or radical resection (12, 30, 40). In these cohorts, survival outcomes were primarily determined by histologic grade, tumor stage, Ki-67 index, and the receipt of systemic chemotherapy rather than by surgical margin status alone, supporting the notion that biliary neuroendocrine carcinoma (NEC) behaves as a systemic disease in many patients at presentation (30, 35, 41). In our series, eight of nine patients achieved R0 resection; however, the median overall survival was only 21 months, and eight patients died during follow-up, further underscoring that tumor biology and treatment strategy may play a more decisive prognostic role than surgical radicality alone.

From a biological perspective, the histologic and molecular characteristics of Biliary NEC closely resemble those of small-cell and large-cell carcinomas. However, the biliary tract itself lacks native neuroendocrine cells, and thus the origin of Biliary NEC remains controversial. The most accepted hypothesis posits that Biliary NEC arises from pluripotent stem cells within the biliary epithelium that undergo neuroendocrine differentiation, or from metaplastic transformation under chronic inflammatory stimulation such as cholelithiasis (29, 34, 42). Another theory proposes that Biliary NEC may develop through “dedifferentiation” of pre-existing adenocarcinoma, in which glandular epithelial cells acquire neuroendocrine features under specific molecular signaling pathways. Regardless of the mechanism, Biliary NEC is characterized by high invasiveness, early local invasion, and a strong propensity for distant metastasis, which collectively explain its aggressive biological behavior and poor clinical outcome.

Previous studies have consistently demonstrated that high-grade histology, elevated Ki-67 index, lymph node metastasis, and lack of systemic chemotherapy are key predictors of unfavorable prognosis in biliary neuroendocrine neoplasms (NENs) (43, 44). Among these factors, Ki-67 is particularly informative because it reflects proliferative activity and correlates with metastatic potential, treatment response, and overall survival in high-grade NENs, and is therefore recommended for risk stratification in clinical practice (45). Lymph node metastasis similarly reflects systemic dissemination and has been associated with significantly reduced postoperative survival in several multicenter and SEER-based analyses. Furthermore, multiple retrospective series have suggested that platinum-based systemic chemotherapy may confer survival benefits, particularly in poorly differentiated biliary neuroendocrine carcinoma (NEC), supporting a treatment paradigm analogous to that used for small-cell lung cancer (46). In our series, four of nine patients had lymph node metastasis and most patients with high Ki-67 indices experienced early death, whereas those who received systemic chemotherapy showed a trend toward longer survival, aligning with previous findings despite the descriptive nature of our cohort.

Nevertheless, several limitations should be acknowledged. First, the small sample size limits the statistical power of our analysis and precludes multivariate survival analysis. Second, detailed data on chemotherapy regimens, cycles, and recurrence patterns were incomplete for some cases. Third, molecular and immunogenomic analyses were not performed, preventing exploration of potential genetic alterations or immune microenvironmental factors influencing disease behavior. Future multicenter collaborations and establishment of dedicated databases for biliary neuroendocrine tumors are warranted to achieve a more comprehensive understanding of this disease. Integrating molecular profiling and immunological characterization may further refine risk stratification and facilitate the development of individualized therapeutic strategies.

## 5 Conclusion

In summary, this study reviewed nine cases of biliary neuroendocrine carcinoma (Biliary NEC) diagnosed and treated at our institution over the past decade. Biliary NEC predominantly affects elderly women and is characterized by nonspecific clinical manifestations, leading to diagnostic challenges. Histopathological and immunohistochemical evaluations remain essential for definitive diagnosis. Surgical resection remains the cornerstone of curative treatment, while systemic chemotherapy may provide additional survival benefit in selected patients.

Despite the limited sample size, our findings highlight the importance of early recognition, aggressive surgical management, and appropriate systemic therapy in improving patient outcomes. Future multicenter studies with larger cohorts and integrated molecular analyses are warranted to elucidate the biological characteristics of Biliary NEC and to develop more precise, individualized therapeutic strategies.

## Data Availability

The original contributions presented in the study are included in the article/supplementary material. Further inquiries can be directed to the corresponding authors.
